# Prevalence of sexual intercourse and risk factors among adolescents in schools in Guinea

**DOI:** 10.4102/jphia.v16i1.1248

**Published:** 2025-08-22

**Authors:** Sidikiba Sidibé, Djiba Diakité, Salifou T. Bangoura, Facely Camara, Mory 1 Kourouma, Hadja F. Camara, Lancinè Dramé, Ansoumane Sidibé, Abdoulaye Diallo, Abdoulaye Sow, Alexandre Delamou, Seni Kouanda

**Affiliations:** 1 Faculty of Sciences and Health Techniques, Gamal Abdel Nasser University, Conakry, Guinea; 2 National Training and Research Centre in Rural Health of Maferinyah, Forécariah, Guinea; 3 Centre de Recherche et de Formation en Infectiologie de Guinée, Conakry, Guinea; 4 Pathfinder International, Conakry, Guinea; 5 Institut de Recherche en Sciences de la Santé (IRSS/CNRST), Ouagadougou, Burkina Faso; 6 Institut Africain de Santé Publique (IASP), Ouagadougou, Burkino Faso; 7 Université Saint Thomas d’Aquin (USTA, Ouagadougou, Burkina Faso

**Keywords:** sexual intercourse, prevalence, contributing factors, adolescents, school, Guinea

## Abstract

**Background:**

Adolescent sexual intercourse in schools is a common phenomenon.

**Aim:**

This study analysed the prevalence and contributing factors of sexual intercourse among adolescents attending school in Guinea.

**Setting:**

This study was conducted in Guinea (Conakry).

**Methods:**

This was an analysis of the secondary data from a survey of adolescents in secondary schools in Guinea. A sex-stratified logistic regression analysis was performed. Adjusted odds ratios (AORs) and its 95% confidence intervals (CIs) were reported. The significance level was set at 0.05.

**Results:**

The overall prevalence of sexual intercourse among adolescents of school-age in Guinea was 35.2%. The sexual intercourse prevalence rate was 42.4% (95% CI: 40.4–44.4) among boys and 32.7% (95% CI: 31.6–33.9) among girls. High school (girls AOR: 1.64; 95% CI: 1.43–1.88 and boys AOR: 1.35; 95% CI: 1.07–1.69), Christian and other (boys AOR: 1.58; 95% CI: 1.22–2.06 and girls AOR: 1.49; 95% CI: 1.27–1.73), having a partner (boys AOR: 7.03; 95% CI: 5.61–6.80 and girls AOR: 6.29; 95% CI: 5.44–7.28), knowledge of family planning (boys AOR: 2.25; 95% CI: 1.83–2.75 and girls AOR: 1.67; 95% CI: 1.47–1.89) and age (boys AOR: 1.50; 95% CI: 1.38–1.63 and girls AOR: 1.74; 95% CI: 1.65–1.84) were the most important contributing factors of sexual intercourse.

**Conclusion:**

Future programmes targeting adolescents should include public health interventions that address these factors related to sexual intercourse to reduce early sexuality in schools.

**Contributions:**

This study will help policymakers make decisions about reducing sexual intercourse among school-attending adolescents.

## Introduction

Adolescence is the period of life between the ages of 10 years and 19 years, and it is crucial for human development, laying the foundations for good health.^1^ Nearly 1.3 billion adolescents (16% of the global population) live in developing countries.^2^ In sub-Saharan Africa, adolescents represent 22% of the population.^3^

During adolescence, the individual undergoes significant biological, psychological and social changes that affect future health and well-being.^1,4^ These profound changes have an impact on adolescents’ sexual behaviour,^5^ including early sexual intercourse.

In developing countries, sexual intercourse exposes 21 million females aged 15 years to 19 years to pregnancy annually, 50% of which is unwanted.^6^ In sub-Saharan Africa, teenage pregnancy rates are twice the global average. Reproductive and sexual problems are responsible for 17% of deaths among adolescents.^3^ In this context, the reproductive and sexual health of adolescents is becoming a public health priority to reduce maternal, neonatal, infant and adolescent mortality.

Adolescent sexual intercourse is a frequent problem and varies according to geographical and socio-cultural contexts. The frequency of reported sexual intercourse was 8.3% among adolescents attending school in Malaysia,^7^ 17.9% among sexually active adolescents attending school in a study involving five sub-Saharan African countries,^8^ 26.2% among adolescents attending school in Tigray, Ethiopia,^9^ 31.66% of adolescents and youth in rural areas of southern Benin had their sexual debut before the age of 15 years^10^ and 42% in the town of Likasi in Congo.^11^

Sexual initiation in adolescents can be influenced by several factors that vary according to geographical and socio-cultural contexts. These factors include socio-demographic characteristics such as gender,^12^ age,^7,8^ parental control and peer influence,^7,8,13,14^ use of psychoactive substances such as tobacco and alcohol^7,8,12^ and exposure to pornographic material.^10,14^

In Guinea, despite the fact that having sex with people under the age of 18 years is punishable by law,^15^ this practice remains a reality in Guinean societies. The results of the Demographic and Health Survey conducted in 2018 indicate that 13% of women and 6% of men aged 15 years to 19 years had their sexual debut before the age of 15 years.^16^ In addition, a survey of schoolchildren aged 10 years to 19 years in the commune of Matoto showed that 45.2% had already had sexual intercourse.^17^

Sexual intercourse among adolescents, often unprotected, can lead to increased risks of sexually transmitted infections, unwanted pregnancy and unsafe abortion.^18,19^ They are also predictive of adolescent use of alcohol, tobacco and other drugs.^20^

Despite the scale of the phenomenon, no nationally representative studies have been carried out on the factors associated with sexual intercourse among school-age adolescents. Analysis of existing secondary data on adolescent sexual and reproductive health in schools will help identify the challenges faced by school-age adolescents and assess the impact of current programmes. It will also enable policymakers to develop targeted strategies to improve adolescent sexual and reproductive health. The aim of this study was to analyse the prevalence and factors associated with sexual intercourse among adolescents attending school in the Republic of Guinea.

## Research methods and design

### Study design

This study is a secondary analysis of data from a cross-sectional survey conducted in 2021 in secondary schools (middle and high schools) in the Republic of Guinea. The survey was conducted as part of a doctoral research project aimed at analysing barriers and facilitators to the adoption of family planning among adolescents and urban youth at risk of unwanted pregnancy.

### Setting

The study covered all middle and high school students in public and private schools in six regions of Guinea (Conakry, Faranah, Kankan, Kindia, Labé and N’zérékoré). All pupils present in the classrooms at the time of the survey were included.

### Study population and sampling strategy

A three-stage sampling of schools (level 1), classrooms (level 2) and students (level 3) was carried out in each administrative region. At the first level, a purposive sampling of public and private schools, middle schools and high schools was carried out in each region. At the second level, classrooms were selected by grade. The classrooms with the highest number of pupils in each grade were selected. At the third level, pupils were selected systematically at random. The survey covered 11 139 students aged 13 years to 24 years. The study population was adolescents aged 13 years to 19 years attending secondary schools in Guinea.

### Data collection and variables

**FIGURE 1:**
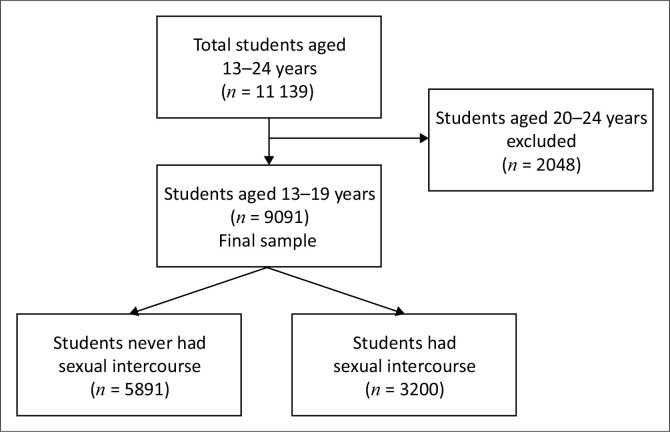
Flow chart for sample size.

Figure 1 shows the sample selection criteria used in our study. The initial sample was made up of students aged 13 years to 24 years. Our analysis was limited to adolescents aged 13 years to 19 years. Two thousand and forty-eight students aged 20 years to 24 years were excluded. The final sample comprised 9091 adolescents aged 13 years to 19 years.

The dependent variable in our study is sexual intercourse. If an adolescent has had sex at least once, the variable is coded ‘yes’, and if an adolescent has never had sex, the variable is coded ‘no’.

The independent variables in our study were age, level of education, type of school, religion (muslim, Christian or others) and region. In addition, adolescents were asked about their knowledge of family planning and having a boyfriend or girlfriend at the time of the survey. The level of education was recorded as middle school (Grades 7–10) and high school (Grades 11–13) and the type of school was classified as public and private. Knowledge of family planning was assessed by various items (having already heard of family planning, knowledge of the definition of family planning, knowledge of the advantages of family planning, knowledge of the disadvantages of family planning, knowledge of at least one contraceptive method and knowledge of sources or place of supply of contraceptive methods).

### Data analysis

Extracted data were exported to Stata 16.0 software for analysis. Data completeness and consistency checked. Analysis was stratified by gender. Descriptive statistics were used to calculate means, standard deviations, numbers and proportions. The prevalence of sexual intercourse was calculated as the ratio of the number of adolescents who had ever had sexual intercourse to the total number of adolescents included in the study. Multicollinearity between explanatory variables was verified using the variance inflation factor (vif). Pearson’s Chi-square test was used to compare differences between categorical variables. Multivariate logistic regression stratified by sex was used to explicate factors associated with sexual intercourse among school-going adolescents. Each variable in the model was adjusted for the remainder of all other variables. Age was analysed as a continuous variable. Adjusted odds ratios (AOR) and 95% confidence interval (CI) were reported. A *p*-value of less than 0.05 was considered statistically significant.

### Ethical considerations

The primary survey was approved by the National Ethics Committee for Health Research (CNERS) under N°045/CNERS/19. Ethical approval was not needed as the data source was conducted after obtaining ethical approval from the Guinea National Ethics Committee. The authors obtained authorisation to use this database.

## Results

### Socio-demographic characteristics of adolescents in schools

A total of 9091 school-going adolescents were selected for this research. The age mean was 17.2 (±1.3) years. Approximately 74% of the adolescents were girls, 58.8% were in junior high school, and 68.6% were studying in the public sector. The Conakry region was the most represented, with 41%. Nearly 61% of teenagers had a boyfriend or girlfriend at the time of the survey and 57% knew about family planning (Table 1).

**TABLE 1:** Characteristics of adolescents in schools, stratified analysis by sex (Guinea, 2021) (*N* = 9091).

Variables	Categories	Global		Girls (*n* = 6781)		Boys (*n* = 2310)	
		*n*	%	*n*	%	*n*	%
Education level	Middle school	5343	58.8	3865	57.0	1478	64.0
	High school	3748	41.2	2916	43.0	832	36.0
Type of school	Public	6238	68.6	4682	69.0	1556	67.4
	Private	2853	31.4	2099	31.0	754	32.6
Religion	Muslim	6938	76.3	5138	75.8	1800	77.9
	Christian and others	2153	23.7	1643	24.2	510	22.1
Region	Conakry	3725	41.0	2897	42.7	828	35.8
	Faranah	1308	14.4	940	13.9	368	15.9
	Kankan	1053	11.6	760	11.2	293	12.7
	Kindia	789	8.7	652	9.6	137	5.9
	Labé	700	7.7	480	7.1	220	9.5
	Nzérékoré	1516	16.7	1052	15.5	464	20.1
Having a boyfriend or girlfriend at the time of the survey	No	3513	38.6	2641	39.0	872	37.7
	Yes	5578	61.4	4140	61.0	1438	62.3
Family planning knowledge	No	3877	42.6	2872	42.3	1005	43.5
	Yes	5214	57.4	3909	57.7	1305	56.5

### Sexual intercourse prevalence among school adolescents

The overall prevalence of sexual intercourse among school-attending adolescents in Guinea was 35.2% (95% CI: 34.2–36.2). Prevalence of sexual intercourse is higher among boys than girls, with 42.4% (40.4–44.4) and 32.7% (31.6–33.9), respectively. The percentage of adolescents who have had sexual intercourse was higher in high school than in middle school in all three models (overall, girls and boys). Adolescents who had a boyfriend or girlfriend at the time of the survey and adolescents who knew about family planning had the highest prevalence of sexual intercourse compared to other modalities in their categories in all three models (overall, girls and boys) (Table 2).

**TABLE 2:** Sex-stratified analysis of the prevalence of sexual intercourse according to the characteristics of adolescents attending school (Guinea, 2021).

Variables	Categories	Global			Girls			Boys		
		*n*	%	*p*	*n*	%	*p*	*n*	%	*p*
Education level	Middle school	1816	34.0	-	1216	31.5	-	600	40.6	-
	High school	1384	36.9	0.004	1005	34.5	0.009	379	45.5	0.021
Type of school	Public	2355	37.7	-	1697	36.2	-	658	42.3	-
	Private	845	29.6	0.000	524	25.0	0.000	321	42.6	0.897
Religion	Muslim	2238	32.3	-	1507	29.3	-	731	40.6	-
	Christian and others	962	44.7	0.000	714	43.5	0.000	248	48.6	0.001
Region	Conakry	1124	30.2	-	821	28.3	-	303	36.6	-
	Faranah	549	42.0	-	346	36.8	-	204	55.2	-
	Kankan	451	42.8	-	301	39.6	-	150	51.2	-
	Kindia	210	26.6	-	154	23.6	-	56	40.9	-
	Labé	150	21.4	-	87	18.1	-	63	28.6	-
	Nzérékoré	716	47.2	0.000	512	48.7	0.000	204	44.0	0.000
Having a boyfriend or girlfriend at the time of the survey	No	431	12.3	-	288	10.9	-	143	16.4	-
	Yes	2769	49.6	0.000	1933	46.7	0.000	836	58.1	0.000
Family planning knowledge	No	992	25.6	-	693	24.1	-	299	29.8	-
	Yes	2208	42.3	0.000	1528	39.1	0.000	680	52.1	0.000

Note: Prevalence of sexual intercourse: Global = 35.2%, 95% CI = 34.2–36.2; girls = 32.7%, 95% CI = 31.6–33.9; boys = 42.4%, 95% CI = 40.4–44.4.

CI, confidence interval.

### Contributing factors of adolescent sex in schools

Table 3 shows the multivariate logistic analysis of contributing factors of sexual intercourse among adolescents attending school in Guinea. Girls in high school were significantly more susceptible to have already had sexual intercourse (AOR: 1.64; 95% CI: 1.43–1.88) than boys (AOR: 1.35; 95% CI: 1.07–1.69). Girls in public school were significantly more prone to have ever had sexual intercourse (AOR: 1.61; 95% CI: 1.40–1.84) than boys (AOR: 0.93; 95% CI: 0.75–1.16). Christian and non-Christian teenagers were significantly more susceptible to have ever had sexual intercourse (boys AOR: 1.58; 95% CI: 1.22–2.06) than Christian teenagers (girls AOR: 1.49; 95% CI: 1.27–1.73). Adolescents who had a girlfriend were significantly more vulnerable to have ever had sexual intercourse (boys AOR: 7.03; 95% CI: 5.61–6.80) than girls who had a boyfriend (AOR: 6.29; 95% CI: 5.44–7.28). Adolescents with knowledge of family planning had a significant probability of ever having had sexual intercourse (boys AOR: 2.25; 95% CI: 1.83–2.75) than girls with knowledge of family planning (AOR: 1.67; 95% CI: 1.47–1.89). For both sexes, region and age were associated with sexual intercourse at school.

**TABLE 3:** Multivariate analysis of contributing factors of sexual intercourse among school adolescents, sex-stratified analysis (Guinea, 2021).

Variables	Girls			Boys			Global		
	AOR	95% CI	*p*	AOR	95% CI	*p*	AOR	95% CI	*p*
**Education level**									
Middle school	Ref.	-	-	Ref.	-	-	Ref.	-	-
High school	1.64	1.43–1.88	< 0.001	1.35	1.07–1.69	0.011	1.58	1.41–1.77	< 0.001
**Type of school**									
Public	1.61	1.40–1.84	< 0.001	0.93	0.75–1.16	0.530	1.35	1.21–1.51	< 0.001
Private	Ref.	-	-	Ref.	-	-	Ref.	-	-
**Religion**									
Muslim	Ref.	-	-	Ref.	-	-	Ref.	-	-
Christian and others	1.49	1.27–1.73	< 0.001	1.58	1.22–2.06	0.001	1.50	1.31–1.71	< 0.001
**Region**									
Conakry	Ref.	-	-	Ref.	-	-	Ref.	-	-
Faranah	2.02	1.68–2.42	< 0.001	2.62	1.94–3.52	< 0.001	2.19	1.88–2.56	< 0.001
Kankan	2.02	1.66–2.44	< 0.001	2.13	1.56–2.91	< 0.001	2.08	1.77–2.45	< 0.001
Kindia	0.89	0.71–1.11	0.306	1.26	0.83–1.92	0.283	0.92	0.76–1.11	0.380
Labé	0.50	0.38–0.66	< 0.001	0.48	0.33–0.69	< 0.001	0.52	0.42–0.65	< 0.001
Nzérékoré	2.14	1.77–2.59	< 0.001	1.53	1.13–2.06	0.006	2.00	1.71–2.35	< 0.001
**Having a boyfriend or girlfriend at the time of the survey**									
No	Ref.	-	-	Ref.	-	-	Ref.	-	-
Yes	6.29	5.44–7.28	< 0.001	7.03	5.61–8.80	< 0.001	6.37	5.64–7.19	< 0.001
**Family planning knowledge**	
No	Ref.	-	-	Ref.	-	-	Ref.	-	-
Yes	1.67	1.47–1.89	< 0.001	2.25	1.83–2.75	< 0.001	1.79	1.61–1.99	< 0.001
Age	1.74	1.65–1.84	< 0.001	1.50	1.38–1.63	< 0.001	1.68	1.60–1.76	< 0.001

AOR, adjusted odds ratio; CI, confidence interval; Ref., reference category.

## Discussion

In this study, we analysed the prevalence of sexual activity among adolescents enrolled in school and its contributing factors. Our study revealed that around a third of school-going adolescents reported ever having had sex. Like our result, earlier studies conducted in sub-Saharan Africa have revealed the variable prevalence of sexual intercourse among school-attending adolescents, with variations according to context. It was 28.3% in Rwanda,^21^ 31.2% in Nigeria,^22^ 41.4% in four Caribbean countries^23^ and 45.2% in a commune in Guinea.^17^ These results show that early sexual intercourse is a common problem, with variations across socio-cultural contexts. The advent of new information and communication technologies, including the Internet, could play a role in the initiation of sexual activity among teenagers.

In this study, the prevalence of sexual intercourse is statistically higher among boys than among girls. This result is comparable to that of Ahanhanzo et al., who found a prevalence of 41.11% among boys and 20.24% among girls.^10^ In Africa, for socio-cultural reasons, boys gain their autonomy earlier than girls, which could justify their early sexual initiation.

In this study, age, educational level, religion, having a partner, family planning knowledge and region were contributing factors of sexual intercourse among adolescents of all sexes at school in Guinea.

As age increased, adolescents were at risk of sexual intercourse. This result is similar to those of other studies.^22,24,25^ Communicating with their children about sexuality can be challenging for some parents, it is regarded as taboo in most African societies.^13,22,24,25^ Another reason is that, as teenagers get older, the influence of their friends on them increases;^24,25^ watching pornographic films and using social networks in this digital age could encourage them to engage in early sexual practices.^25^

Adolescent high school students were more at risk of sexual intercourse than middle school students in our study. The level of education is closely linked to age. This result is corroborated by several studies that have shown that higher levels of education are associated with more sexual intercourse among adolescents.^25,26,27^ What’s more, the lack of a formal sex education programme in secondary schools in Guinea could have an adverse effect on adolescents’ sexual health. In a study carried out in rural areas of South Africa, some participants reported that they resorted to sex as a distraction because of a lack of entertainment venues.^24^ The results of another study showed that students in schools that implemented a sex education programme were less susceptible to early sexual intercourse than students from comparable schools.^28^

In our study, Muslim adolescents were less at risk of early sexual intercourse. This result is similar to some studies^27,29^ but different from others.^25^ In a study carried out in similar contexts, this correlation was explained by faith in the Muslim religion, reading the Koran and praying regularly, which could prevent early sexual activity in unmarried adolescents.^30^

In our study, having a partner was a risk factor for sexual intercourse among adolescents. Having a boyfriend could lead most teenagers into early sexual practices. Some research has shown that cohabitation or marriage increases the likelihood of early sexual intercourse.^31,32^

Knowledge of family planning was a risk factor for teenage sex. The reason could be that knowledge of contraceptive methods helps prevent sexually transmitted infections and early pregnancies. However, adolescents’ knowledge of contraceptive methods and their importance could lead them into early sexual practices.

Adolescents living in the Nzérékoré and Kankan regions were more at risk of sexual intercourse. In contrast, those in the Labé region were less at risk than adolescents in the Conakry region.

Our study has certain limitations. Because of its transversal nature, it does not allow us to establish a causal link between sexual intercourse among adolescents and the associated factors. Because of the taboo nature of sexuality, the prevalence of sexual intercourse may be underestimated as responses are self-reported. In addition, the influence of other factors could not be assessed in this study, such as parental control and consumption of psychoactive substances such as tobacco and drugs. However, this study is the first to be conducted on a nationally representative sample. In addition, the results could help decision-makers to implement effective actions reduce the prevalence of sexual intercourse among adolescents in schools and serve as a basis for further studies.

## Conclusion

Sexual intercourse is common among teenagers at school in Guinea. The study revealed that around a third of school-going adolescents reported having already had sexual intercourse. Age, education level, religion, having a partner, knowledge of family planning and region of residence were factors associated with sexual intercourse.

This study provides evidence that could help policymakers in the decision-making process concerning the earliness of sexual intercourse. The integration of sex education programmes in schools could be an opportunity to educate adolescents about the consequences of early sexuality. Future research could use a mixed approach, integrating issues related to the use of psychoactive substances such as tobacco and drugs, as well as the influence of parents, peers and teachers.
